# MIER3 suppresses colorectal cancer progression by down-regulating Sp1, inhibiting epithelial-mesenchymal transition

**DOI:** 10.1038/s41598-017-11374-y

**Published:** 2017-09-08

**Authors:** Man Peng, Yukun Hu, Wen Song, Shiyu Duan, Qiong Xu, Yanqing Ding, Jian Geng, Jun Zhou

**Affiliations:** 1 Department of Pathology, Nanfang Hospital, Southern Medical University, Guangzhou, 510515 China; 2 Department of Pathology, School of Basic Medical Sciences, Southern Medical University, Guangzhou, 510515 China; 3 Guangdong Provincial Key Laboratory of Molecular Oncologic Pathology, Southern Medical University, Guangzhou, 510515 China

## Abstract

Mesoderm induction early response 1, family member 3 (MIER3) has recently been identified as a potential cancer susceptibility gene. However, the expression pattern and the role of MIER3 in the progression of colorectal cancer (CRC) have not yet been well characterized. Here, we reported that MIER3 was significantly reduced in human primary colorectal cancer and was associated with CRC metastasis and poor prognosis. Moreover, the up-regulation of MIER3 expression significantly inhibited CRC cell proliferation, migration and invasion *in vitro* and repressed tumor growth and metastasis *in vivo*. In contrast, down-regulation of MIER3 could promote the aggressive behaviors of CRC cells. Furthermore, our study showed that MIER3 inhibited cell proliferation and invasion partially via reduction of Sp1 and subsequent suppression of epithelial-mesenchymal transition (EMT). In conclusion, our data suggested that MIER3 plays a potential tumor suppressor role in CRC progression and may be a potentially valuable clinical prognostic marker of this disease.

## Introduction

Colorectal cancer (CRC) is one of the most common cancers in the world and is considered the third leading cause of cancer-related death^1^. In China, the incidence of CRC is continually increasing. Despite advances in diagnostic and therapeutic techniques^2^, the prognosis of CRC patients with distant metastases remains poor^3^, and the molecular underpinnings of CRC metastasis are still unclear. Thus, it is urgent to identify important molecules in CRC progression, which may be used to develop new diagnostic strategies and drugs targeting these markers.

Mesoderm induction early response 1, family member 3 (MIER3) belongs to the MIER family, which includes three members: MIER1, MIER2 and MIER3^4^. Much of the work to date has focused on MIER1, which has been shown to function as a transcriptional repressor^5^. The biological characteristics and functions of MIER2 or MIER3 are not yet known, both are predicted to be nuclear proteins^6, 7^ and to associate with HDAC1 and/or HDAC2^8–11^. In addition, recent studies have found that MIER3 has a high mutation frequency in hypermutated colorectal tumors^12^, and it is considered a candidate breast cancer susceptibility gene that may play a role in tumorigenesis^13^. However, the role and mechanism of MIER3 in CRC are not yet completely understood. In this study, we detected MIER3 expression in clinical CRC tissue samples and CRC cell lines and investigated the effects of aberrant MIER3 expression on the cellular behaviors of CRC cells *in vitro* and on tumor growth and metastasis *in vivo* and the possible molecular mechanisms underlying the functions of MIER3.

## Results

### Expression of MIER3 was down-regulated in CRC and associated with metastasis

**Figure 1:**
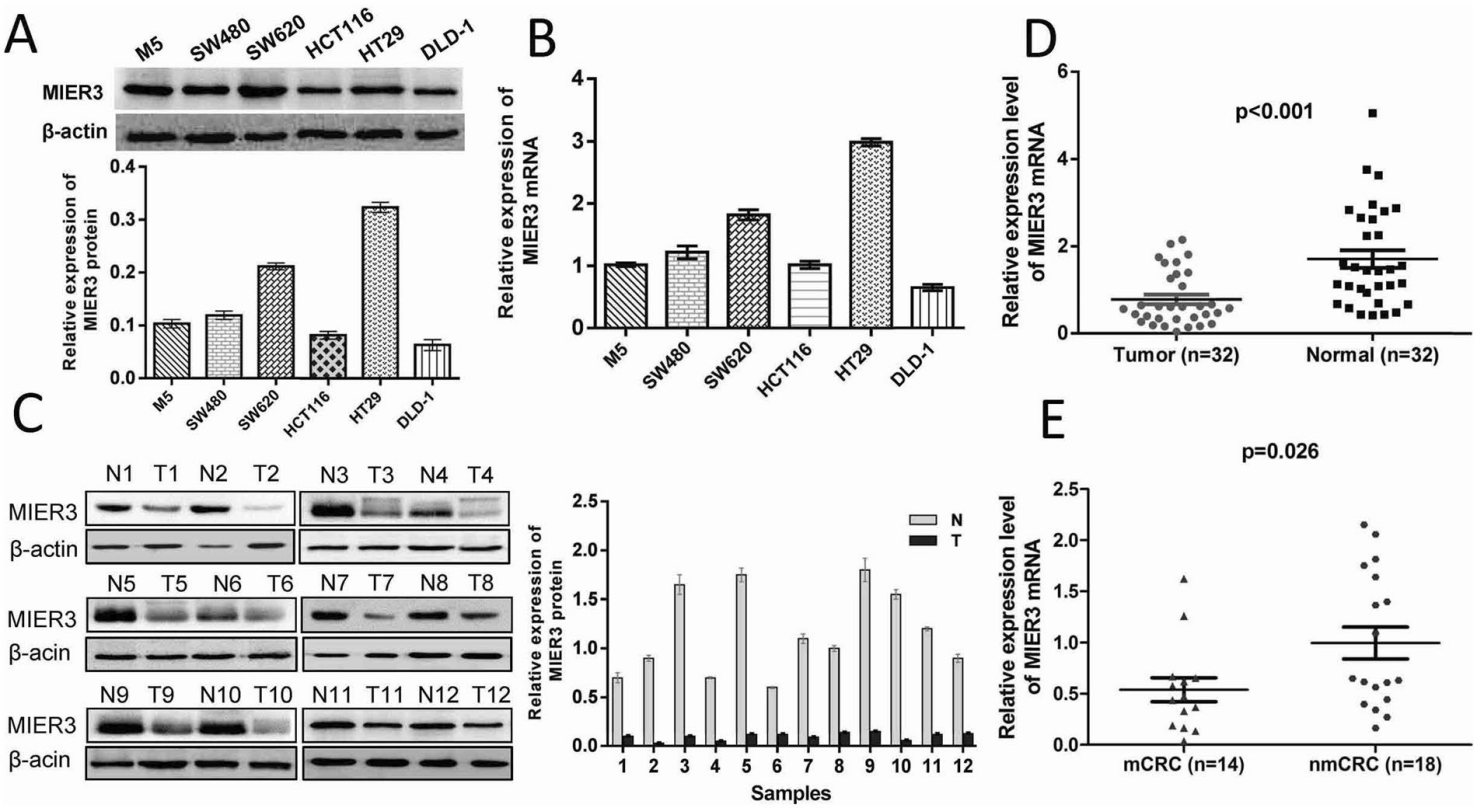
Expression of MIER3 mRNA and protein in CRC cells and colorectal tumor tissue samples. (**A**) Expression of MIER3 protein by western blotting (above). The protein expression levels were quantified by comparing the gray level of each band using Quantity One Software (below). Data are the means ± SD from 3 parallel experiments. (**B**) Expression of MIER3 mRNA by real-time PCR in six CRC cell lines. Expression levels of MIER3 mRNA were normalized with β-actin mRNA levels. Error bars represent the mean ± SD calculated from 3 parallel experiments. (**C**) Expression of MIER3 protein in each of the primary CRC (T) and adjacent noncancerous tissues (N) paired from the same patient by western blotting (left) (n = 12). The protein expression levels were quantified by comparing the gray level of each band using Quantity One Software (right). Error bars represent the mean ± SD calculated from 3 parallel experiments. (**D**) Expression levels of MIER3 mRNA by real-time PCR in paired CRC and adjacent normal tissues (n = 32). Expression levels of MIER3 mRNA were normalized with β-actin mRNA levels. Error bars represent the mean ± SD calculated from 3 parallel experiments. (**E**) MIER3 expression in CRC tissues with or without metastases. nmCRC denotes CRC tissues without metastases; mCRC denotes CRC tissues with lymph node metastases.

Real-time PCR and western blotting analysis were performed to detect the expression of MIER3 in 6 CRC cell lines: M5, SW480, SW620, HCT116, HT29, and DLD-1. Our results revealed that MIER3 was differentially expressed in all six colorectal cancer cell lines. Compared with the MIER3 expression level in M5 cells, the MIER3 expression level was significantly higher in HT29 and SW620 cells and significantly lower in HCT116 and DLD1 cells (Fig. 1A,B). Furthermore, comparative analyses indicated that MIER3 protein expression was significantly down-regulated in the 12 examined tumor samples paired with adjacent non-neoplastic mucosa tissues (Fig. 1C). The expression of MIER3 mRNA in 32 CRC tissues was significantly lower than that in corresponding normal tissues (*p* < 0.001; Fig. 1D), and the expression of MIER3 in patients with lymph node metastasis was significantly lower than that in patients without metastasis (*p* = 0.026; Fig. 1E). This association indicated that MIER3 might have a pivotal role in CRC metastasis.

### Down-regulation of MIER3 is associated with progression and poor prognosis in CRC

**Figure 2:**
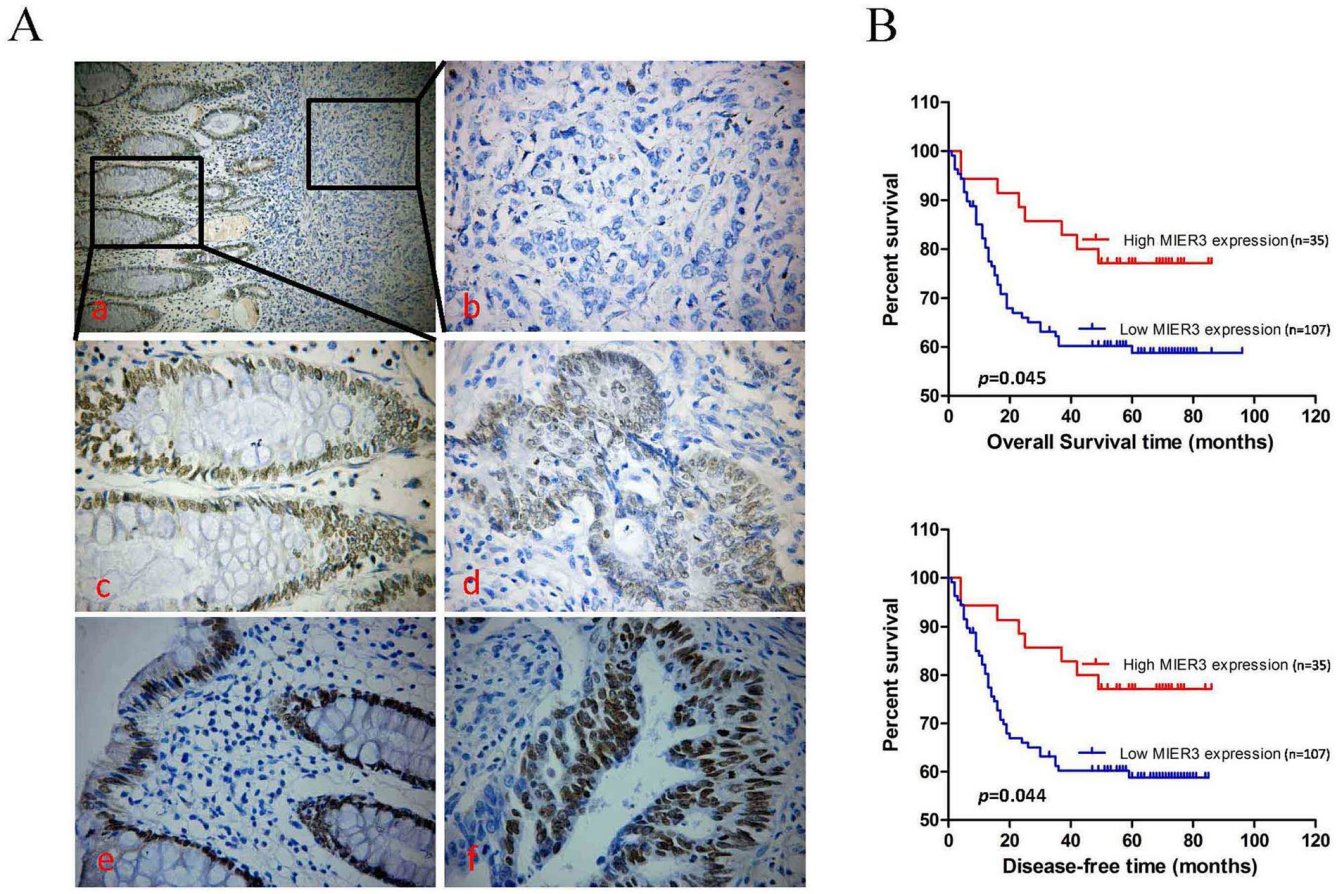
MIER3 is down-regulated in paraffin-embedded clinical CRC tissues and is a prognostic factor for poor overall and disease-free survival in CRC patients. (**A**a–c) Representative images of MIER3 expression in normal intestinal epithelium and CRC specimens as examined by IHC. Negative staining for MIER3 in a tumor tissue sample and high expression of MIER3 in its normal mucosal counterpart from the same patient were observed in one field (original magnification 200×) or two independent magnification fields (original magnification 400×). (**d**) Positive expression of MIER3 in colorectal tumor tissue samples (original magnification 400×). (**e**,**f**) High expression of MIER3 in normal intestinal epithelial cells and CRC cells (original magnification 400×), (**B**) Kaplan–Meier survival analysis of (above) overall and (below) disease-free survival duration in all patients according to MIER3 expression. The log-rank test was used to calculate *p* values.

To explore whether MIER3 expression levels are associated with the clinicopathological factors of CRC, we measured MIER3 expression in a large cohort of 142 archived paraffin-embedded CRC and normal colon tissues using IHC. The results showed that MIER3 protein was located in the nuclei of benign and malignant epithelial cells (Fig. 2A). Additionally, we found that MIER3 was highly expressed in 37 of 62 (59.7%) normal colon mucosa samples obtained from bowels without tumors. In comparison, we observed high levels of MIER3 expression in 35 of 142 (24.6%) colorectal tumor tissue samples. We further analyzed the association between the level of MIER3 expression and clinicopathologic features of CRC by χ2 test and Fisher’s exact test. As summarized in Table 1, the expression of MIER3 was significantly associated with differentiation (*p* = 0.038), clinical stage (*p* = 0.035), T classification (*p* = 0.029), lymph node metastasis (*p* = 0.029), and distant metastasis (*p* = 0.022) in patients with CRC. However, it was not associated with other clinicopathological features, including patient age, sex, tumor site, and tumor size (*p* > 0.05; Table 1). In addition, the results of Kaplan-Meier survival analysis with the log-rank test indicated that the expression of MIER3 in CRC was significantly correlated with the overall survival (log rank = 4.022, *p* = 0.045; Figure B above) and disease-free survival (log rank = 4.039, *p* = 0.044; Figure B below) of CRC patients. Low levels of MIER3 expression were associated with poor survival. Cox regression analyses revealed that in this study, distant metastasis and MIER3 expression were recognized as independent prognostic factors (*p* < 0.05; Table 2).

**Table 1:** Relationship between clinicopathological features and expression of MIER3.

Characteristics	n	MIER3 expression		
		low (%)	high (%)	*p* value
**Gender**				
Male	88	65	23	0.599
Female	54	42	12	
**Age (years)**				
<50	67	52	15	0.555
≥50	75	55	20	
**Tumor site**				
Proximal colon	42	32	10	0.925
Distal colon	35	27	8	
Rectum	65	48	17	
**Tumor size (cm in diameter)**				
<5	48	37	11	0.732
≥5	94	70	24	
**Tumor differentiation**				
Good	14	10	4	**0.023**
Moderate	104	74	30	
Poor	24	23	1	
**Clinical stage**				
1	15	8	7	0.035
2	53	37	16	
3	32	25	7	
4	42	37	5	
**T stage**				
1~2	23	13	10	0.029
3	98	75	23	
4	21	19	2	
**Lymph node state**				
Positive	59	50	9	0.029
Negative	83	57	26	
**Distant metastasis**				
Positive	42	37	5	0.022
Negative	100	70	30

**Table 2:** Summary of overall survival analyses by univariate and multivariate COX regression analysis.

	Univariate analysis			Multivariate analysis		
	*p* value	HR	95% Confidence interval	*p* value	HR	95% Confidence interval
Gender	0.619	0.876	0.519–1.477			
Age	0.562	0.862	0.522–1.424			
Tumor site	0.837	0.969	0.720–1.304			
Tumor size	0.077	1.692	0.944–3.032			
Tumor differentiation	0.003	2.158	1.306–3.568	0.271	1.316	0.807–2.147
Lymph node state	0.007	1.997	1.207–3.304	0.374	1.272	0.748–2.161
Distant metastasis	<0.001	5.502	3.281–9.226	<0.001	4.446	2.578–7.668
MIER3 expression	0.004	0.332	0.158–0.699	0.046	0.461	0.251–0.987

### Overexpression of MIER3 suppresses the proliferation, invasion and migration of CRC cells *in vitro*

**Figure 3:**
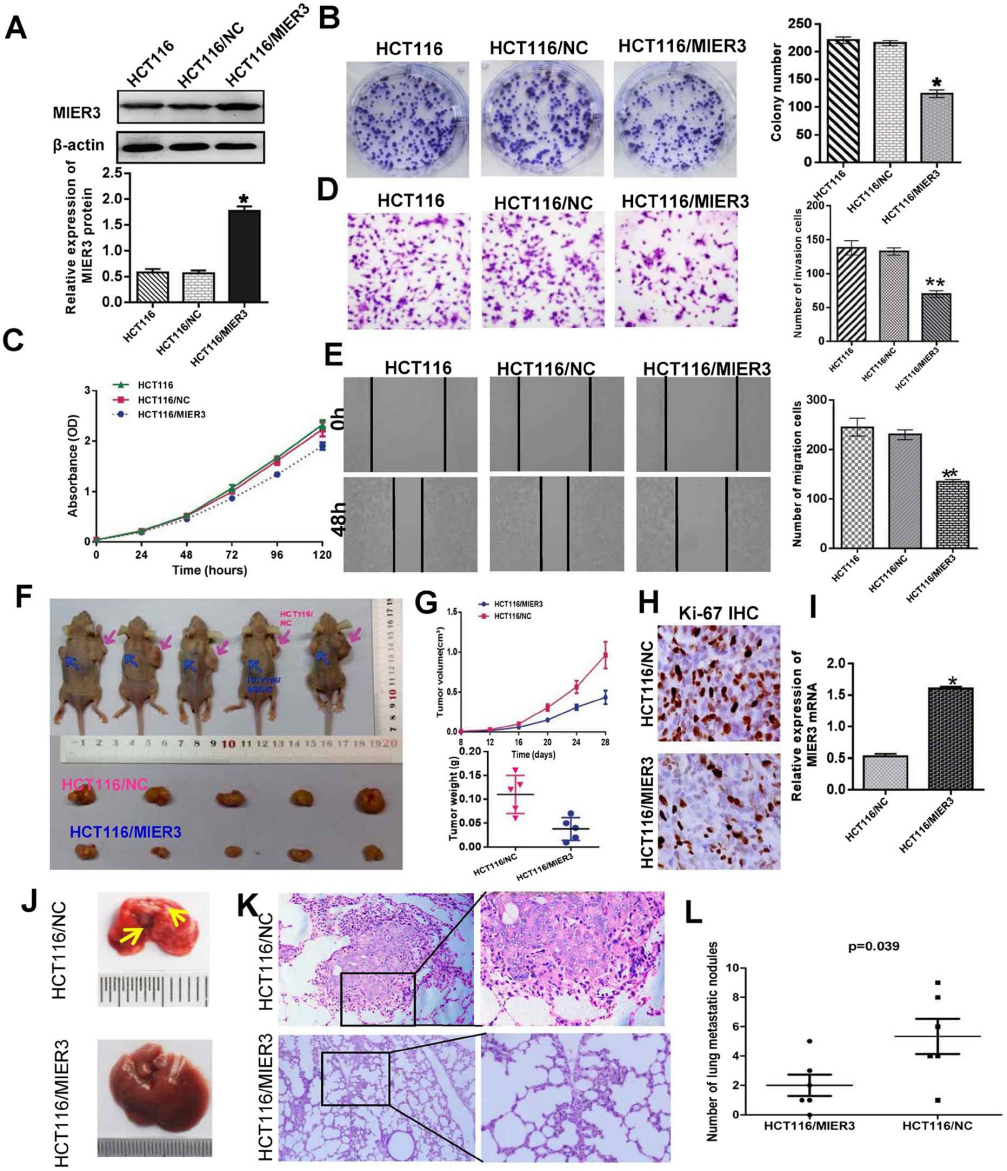
Overexpression of MIER3 inhibited the aggressive phenotype of CRC *in vitro* and *in vivo*. (**A**) Ectopic expression of MIER3 in HCT16 cells analyzed by western blotting. (**B**,**C**) Ectopic expression of MIER3 inhibits cell proliferation as determined by colony formation (**B**) and MTT assays (**C**) (*p* < 0.05). (**D**, **E**) Effects of MIER3 overexpression on the migration and invasion of HCT116 cells as determined by Matrigel invasion and scratch wound-healing assay. Each bar represents the mean ± SD of three independent experiments. (**F**) MIER3-overexpressing HCT16 cells and mock cells injected subcutaneously into nude mice. At 28 days after subcutaneous injection, HCT16/MIER3 and HCT16/mock cells produced primary tumors (upper); a representative sample of the tumors that formed is shown (lower). (**G**) Tumor growth curve. Each data point represents the mean tumor volume ± SD (upper) and scatter plots of the weights of tumors derived from mice injected with HCT16/MIER3 and HCT16/mock cells at 28 days after subcutaneous injection (upper). (**H**) Immunohistochemistry showed that overexpression of MIER3 reduced the proliferation index by Ki67 staining (200× ). (**I**) Real-time PCR was used to test MIER3 expression in xenograft tumors formed from HCT16/MIER3 and HCT16/mock cell injections. Error bars represent the mean ± SD. (**J**,**K**) The whole-body images (**J**) and histological images (**K**) of metastatic nodules in the lungs. (**L**) Lung metastatic nodules in individual mice were counted under the microscope. *p < 0.05, **p < 0.001.

To explore the potential biological functions of MIER3 in CRC, we established a stable MIER3 overexpression cell line, HCT116/MIER3, and a control cell line, HCT116/NC, by infecting cells with recombinant lentivirus. Increased expression of MIER3 upon infection in the cell line HCT116 was confirmed by western blot (*p* < 0.001; Fig. 3A). The results of colony formation assays and CCK-8 assays showed that MIER3 overexpression inhibited the proliferation of HCT116 cells compared with that of the control (*p* < 0.05; Fig. 3B,C). We also examined the effect of MIER3 on CRC cell migration and invasion potency using wound-healing and Matrigel invasion assays. Compared with the control, the cell line HCT116/MIER3 showed a significant reduction in invasive tendency in the Matrigel invasion assay (*p* < 0.001; Fig. 3D). Wound-healing assays also illustrated that overexpression of MIER3 in CRC cells inhibited cell migratory capacity (*p* < 0.001; Fig. 3E).

### Overexpression of MIER3 inhibits tumor growth and metastasis *in vivo*

The effect of MIER3 on tumor growth was assessed *in vivo*. HCT116/MIER3 and HCT116/NC cells were injected into nude mice. All mice developed xenograft tumors at the injection site. As shown in Fig. 3F–I, tumors formed from HCT116/MIER3 cells grew more slowly than those formed from HCT116/NC cells (n = 5; *p* < 0.01). Moreover, by immunohistochemical staining for Ki-67, we found that the proliferation index was reduced in tumor tissues formed from HCT116/MIER3 cells compared with those formed from HCT116/NC cells (*p* < 0.05). The effect of MIER3 on metastasis was assessed 8 weeks after injection. We examined the number and size of tumor metastatic nodules in the lung under a microscope. As shown in Fig. 3J,L, the number of pulmonary metastatic nodules was obviously decreased in the HCT116/MIER3 group compared with that in the HCT116/NC group (*p* = 0.039).

### Inhibition of endogenous MIER3 promotes the proliferation, invasion, and migration of CRC cells *in vitro*

**Figure 4:**
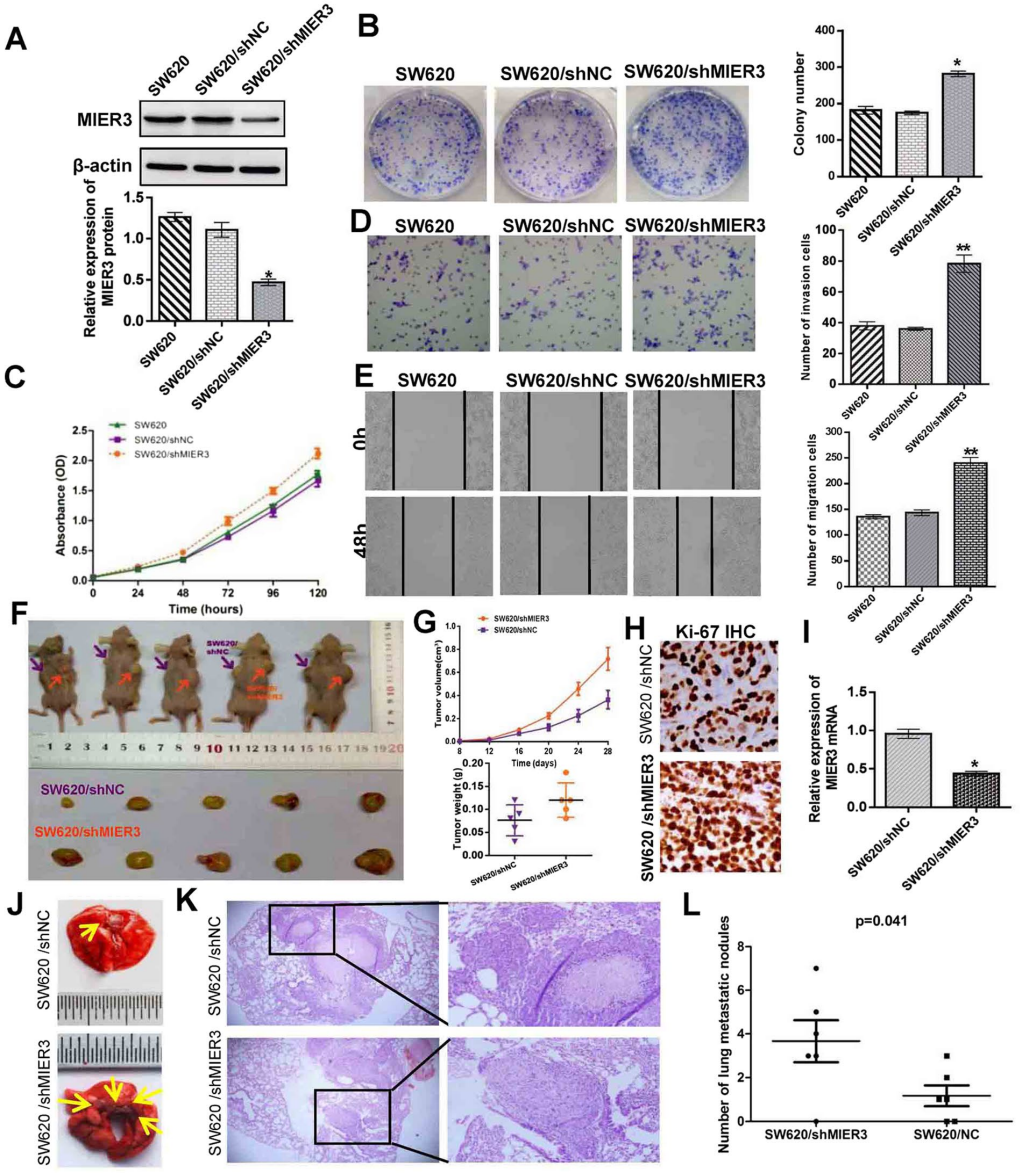
RNAi-silencing of MIER3 promotes the aggressive phenotype of CRC *in vitro* and *in vivo*. (**A**) RNAi-silencing of MIER3 in shRNA-transduced stable SW620 cells. (**B**,**C**) Silencing of endogenous MIER3 promoted cell growth as determined by colony formation assays (**B**) (*p* < 0.05) and MTT assays (**C**,) (*p* < 0.05). (**D**, **E**) Effects of decreased MIER3 on the migration and invasion of SW620 cells as determined by Matrigel invasion and scratch wound-healing assay. Each bar represents the mean ± SD of three independent experiments. (**F**) MIER3 knockdown SW620 cells and mock cells injected subcutaneously into nude mice. At 28 days after subcutaneous injection, SW620/shMIER3 and SW620/mock cells produced primary tumors (upper); a representative sample of the tumors that formed is shown (lower). (**G**) Tumor growth curve. Each data point represents the mean tumor volume ± SD (upper) and scatter plots of the weights of tumors derived from mice injected with SW620/shMIER3 and SW620/mock cells at 28 days after subcutaneous injection (upper). (**H**) Immunohistochemistry showed that MIER3 knockdown increased the proliferation index by Ki67 staining ( ×200). (**I**) Real-time PCR was used to test MIER3 expression in xenograft tumors formed from injections with SW620/shMIER3 and SW620/mock cells. Error bars represent the mean ± SD. (**J**,**K**) The whole-body images (**J**) and histological images (**K**) of metastatic nodules in the lungs. (**L**) Lung metastatic nodules in individual mice were counted under a microscope. *p < 0.05, **p < 0.001.

To further confirm the impact of MIER3 on the proliferation, invasion, and migration of CRC cells, we knocked down endogenous MIER3 in SW620 CRC cells using shRNAs specifically targeting MIER3 (*p* < 0.001; Fig. 4A). Colony formation assays and MTT assays demonstrated that down-regulation of MIER3 caused obviously increased proliferation in SW620 cells compared to control cells (*p* < 0.05; Fig. 4B,4C). Meanwhile, Matrigel invasion and wound-healing assays confirmed that knock down of MIER3 increased the migration and invasion of SW620/shMIER3 cells compared with that of control cells (*p* < 0.001; Fig. 4D,E). Thus, these data revealed that MIER3 down-regulation could promote CRC cell proliferation, invasion and migration *in vitro*.

### Inhibition of endogenous MIER3 promotes tumor growth and metastasis *in vivo*

To further confirm the effects of MIER3 on tumor growth in nude mice, SW620/shMIER3 cells and SW620/NC cells were used for xenograft tumor assays. The results are shown in Fig. 4F–I. Inhibition of endogenous MIER3 in SW620 cells significantly increased tumor growth and the expression of Ki-67 compared with that of the control. The effects of endogenous MIER3 inhibition on metastasis was also assessed after the nude mice had been injected with the cancer cells through the tail vein. Compared with the control group, we found that significantly more and larger tumors nodules were formed in the lungs of mice from the SW620/shMIER3 group than lungs from control mice (Fig. 4J,L). These results indicate that MIER3 down-regulation could promote tumor growth and metastasis *in vivo*.

### MIER3 directly reduces Sp1 levels and inhibits epithelial-mesenchymal transition

**Figure 5:**
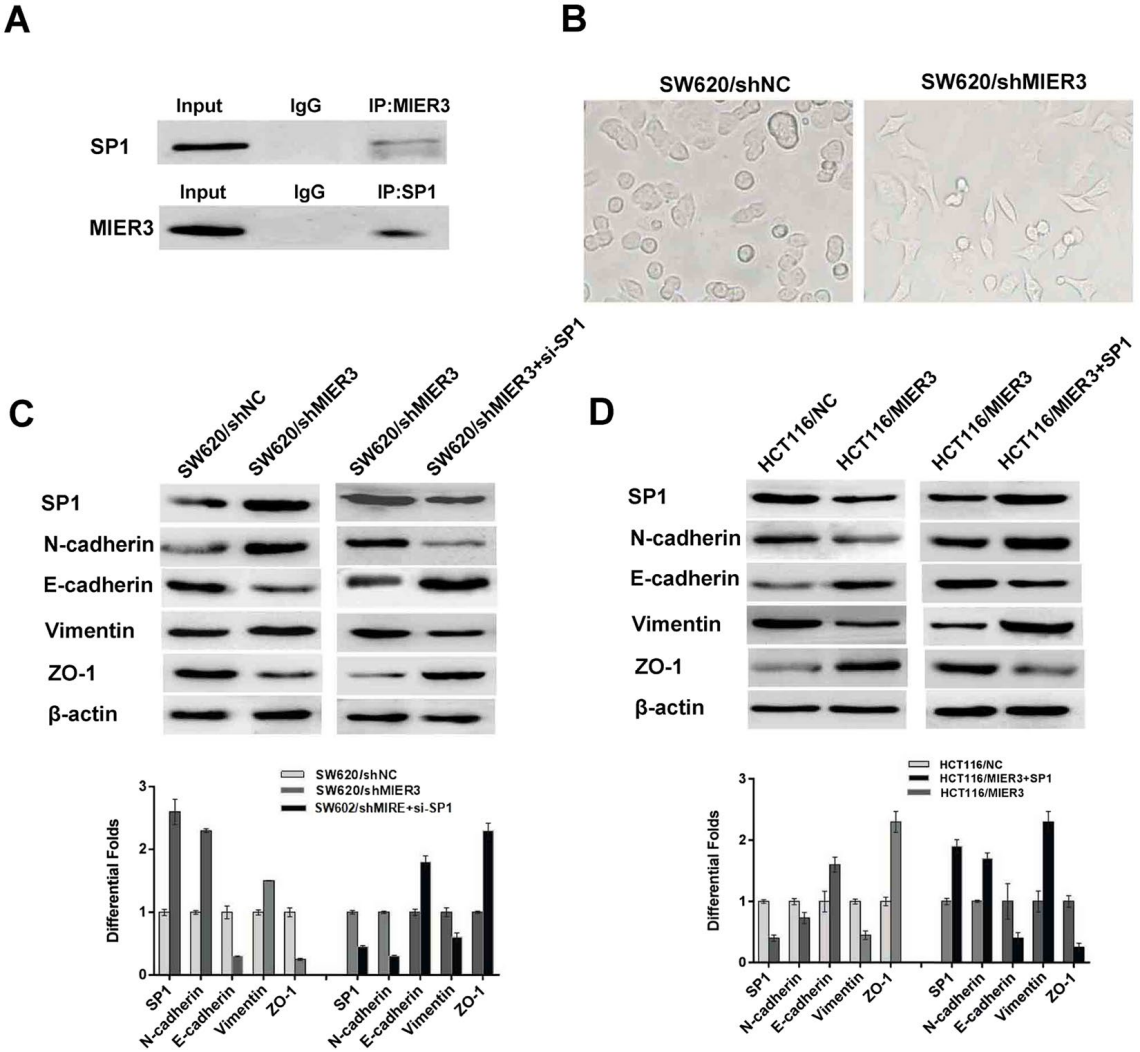
MIER3 is associated with Sp1 and promotes EMT in CRC cells. (**A**) MIER3 co-immunoprecipitates with Sp1 in CRC cells. Lysates from SW620 cells were immunoprecipitated with MIER3 antibody or control IgG and detected with Sp1 antibody on a western blot and then immunoprecipitated with Sp1 antibody or control IgG and detected with MIER3 antibody on a western blot. (**B**) The spindle cell phenotype of SW620/shMIE3 cells and the epithelial phenotype of CRC cells from the SW620/shNC control group showing epithelial-to-mesenchymal transition (EMT) induced by decreased MIER3 expression. (**C**) MIER3 knockdown led to increased Sp1 expression and induced hallmarks of EMT, including decreased E-cadherin and ZO-1 and the accumulation of vimentin and N-cadherin in CRC cells. In contrast, down-regulation of Sp1 led to decreased E-cadherin and ZO-1 expression and increased vimentin and N-cadherin expression. (**D**) MIER3 overexpression led to decreased Sp1 expression and induced the accumulation of E-cadherin, ZO-1 and the decreased expression of vimentin, and N-cadherin in CRC cells. While up-regulation of Sp1 led to increased E-cadherin and ZO-1, it inhibited vimentin and N-cadherin expression. Error bars represent the mean ± SD.

We next explored the possible mechanisms by which MIER3 inhibits the proliferation and invasion of CRC cells. Co-immunoprecipitation analysis revealed a protein-protein interaction between MIER3 and Sp1, indicating that endogenous human MIER3 physically associates with Sp1 (Fig. 5A). More intriguingly, comparison of the modified CRC cell line SW620/shMIER3 and its control group showed that silencing endogenous MIER3 induced non-invasive epithelial cells to acquire a mesenchymal, spindle cell phenotype, suggestive of epithelial-mesenchymal transition (EMT) (Fig. 5B). We therefore further investigated the expression of markers associated with EMT. Western blot analyses showed that down-regulation of MIER3 resulted in an increase in the expression of vimentin and N-cadherin. Meanwhile, overexpression of MIER3 led to substantial up-regulation of E-cadherin and ZO-1 expression, which is associated with an epithelial phenotype. Furthermore, we found that the expression level of Sp1 was significantly decreased in HCT116/MIER3 cells. In contrast, knockdown of MIER3 in SW620 cells dramatically up-regulated the expression of Sp1. Moreover, we conducted rescue assays to determine whether Sp1 was involved in the MIER3 silencing-associated increases in colorectal cancer cell proliferation and invasion. We performed a rescue experiments by transfecting SW620/shMIER3 cells with si-Sp1 or transfecting HCT116/MIER3 cells with pcDNA3.1-Sp1. Inhibition of endogenous Sp1 could partially rescue the MIER3-silencing-induced hallmarks of EMT. In contrast, the expression of exogenous Sp1 could partially rescue MIER3-mediated inhibition of EMT (Fig. 5C–D). Taken together, these results indicated that Sp1 could abrogate the MIER3-induced inhibition of EMT in CRC, which further confirms that MIER3 suppresses CRC progression, at least in part, by directly reducing Sp1 and subsequently suppressing EMT.

## Discussion

MIER3 is an essentially unresearched gene that encodes a member of the MIER family, which includes MIER1, MIER2, and MIER3^14^. However, in 2012, it was shown that MIER3 probably plays an important role in tumorigenesis because it was described as a candidate breast cancer susceptibility gene^13^ and a highly mutated gene in hypermutated colorectal tumors^12^. Subsequent studies found that the expression of MIER3 mRNA in colorectal cancer tissues is down-regulated but only suggested that MIER3 mRNA expression was down-regulated in CRC and not related to clinical data; there was also a lack of further functional studies^15^, leaving the exact role and mechanism of MIER3 in cancer still unknown. Because mutations in tumor-associated genes often lead to gene expression disorders and dysfunction, we first evaluated the relationship between MIER3 expression and clinicopathological features of CRC. The results showed that the expression of MIER3 was down-regulated in CRC at mRNA and protein levels compared to normal intestinal epithelial tissues. Low expression levels of MIER3 were significantly associated with advanced clinical disease stage, tumor T classification and poor survival in patients. This result implies that down-regulation or loss of MIER3 protein expression might serve as a biomarker to identify patients with more aggressive CRC and poorer clinical outcomes. However, previous studies showed that MIER3 transcription levels are significantly higher in breast cancer compared to normal breast tissue^13^, which differs from our results in colorectal cancer. These different findings suggest that the specificity and complexity of MIER3’s function and related mechanisms vary in different tumor types.

To highlight the function of down-regulation of MIER3 in CRC, we further explored the critical role of MIER3 in the progression of CRC by knock down and overexpression experiments. Our results revealed that the down-regulation of MIER3 expression could significantly promote CRC cell proliferation, migration and invasiveness *in vitro*. In contrast, increasing the expression of MIER3 could inhibit the aggressive behaviors of CRC cells. Furthermore, reducing the expression of MIER3 could promote tumorigenesis and lung metastasis in a murine model. Taken together, these findings suggested that MIER3 might serve as an anti-oncogene and play an inhibitory role in CRC development and progression.

The molecular mechanisms underlying MIER3-mediated biological behaviors are still unclear, as MIER3 encodes an uncharacterized member of the MIER family of proteins. While the gene product of MIER1 is a fibroblast growth factor (FGF)-activated transcriptional regulator, studies recently have shown that MIER1 is associated with breast cancer progression^16^ and may control transcription using several distinct mechanisms, including recruitment of HDAC1^17^, inhibition of the histone acetyltransferase activity of CBP and displacement of Sp1 from its associated sites in the promoters of target genes^18, 19^.

Sp1 is a member of the Sp general transcription factor family^20^ and plays an important role in both transcription initiation and activation^21^. In recent years, accumulating evidence has shown that Sp1 regulates the expression of various genes that are important in tumorigenesis, such as genes related to cell proliferation, differentiation, apoptosis, drug resistance and metastasis^22^. Moreover, it has been reported that Sp1 is required for TGF-β-induced and Smads-mediated epithelial-to-mesenchymal transition (EMT) via transcriptional induction of vimentin^23^. In addition, Sp1 can up-regulate E-cadherin repressors such as ZEB1/2, SNAIL, and TWIST1, which subsequently induce EMT^24^.

Human MIER3 and MIER1 are structurally similar; 54% of their amino acids are identical based on BLAST analysis^25^, and they both contain several domains important for their role in regulating transcription. These domains include an N-terminal acidic region^26^, an ELM2 domain and a SANT domain^27, 28^. MIER3 and MIER1 are highly conserved in structure, suggesting that these two proteins may share similar functions in some respects. Because previous studies suggested that MIER1 could physically interact with Sp1^21^ and because we had observed MIER3-mediated reduction of migration and invasion in CRC, in the present study, we attempted to explore whether MIER3 could physically interact with Sp1 to affect the expression of EMT-associated proteins and induce EMT in CRC. The results showed that MIER3 might interact with Sp1 and that inhibition of endogenous Sp1 resulted in an increase in the epithelial markers E-cadherin and ZO-1 and a decrease in the mesenchymal markers vimentin and N-cadherin. However, the details of the underlying mechanism need further investigation.

In conclusion, our findings suggested that down-regulation of MIER3 was significantly associated with CRC progression and poor survival in patients. All the functional experiments also confirmed MIER3 as a tumor suppressor in the progression of CRC. The increased down-regulation of MIER3 in CRC patients with invasion and metastasis suggested that MIER3 might be a significant biomarker for CRC progression. However, the detailed molecular mechanisms of MIER3 in CRC progression still need to be further investigated.

## Methods

### Cell culture

Human embryonic kidney 293 T cells and the human CRC cell lines DLD-1, HT29, HCT116, SW480, and SW620 were obtained from a cell bank at the Chinese Academy of Sciences (Shanghai, China). All cells were authenticated by short tandem repeat (STR) profiling after receipt and were propagated for less than 6 months after resuscitation. M5 is a subclone that we had established in previous studies with enhanced liver metastatic abilities^29, 30^. All CRC cell lines were cultured in RPMI 1640 medium (Gibco, Gaithersburg, MD, USA) with 10% fetal bovine serum (HyClone, Logan, USA) and 100 U/ml penicillin/streptomycin (Gibco). These cell lines were maintained in a humidified chamber containing 5% CO_2_ at 37 °C.

### Tissue preparation

Fresh and formalin-fixed, paraffin-embedded colorectal tumor tissue samples were obtained from patients at Nanfang Hospital at Southern Medical University (Guangzhou, China). None of these patients received any preoperative chemotherapy or radiotherapy. In total, 32 cases of fresh CRC tissue were freshly frozen in liquid nitrogen and stored at −80 °C until further use. Additionally, 142 cases of archived, formalin-fixed, paraffin-embedded CRC tissue samples were collected and used in the clinicopathological and prognostic investigation of MIER3. A comprehensive set of clinicopathological data was recorded, including age, gender, size of primary tumor, tumor differentiation, tumor T stage, lymph node metastasis, and distant metastasis. The stage of disease was determined according to the tumor size, lymph node, and metastasis (pTNM) classification system. Complete follow-up, ranging from 1–96 months, was available for the cohort of 142 patients, and the median survival was 56 months. The study was approved by the Ethics Committee of Southern Medical University, and all aspects of the study comply with the Declaration of Helsinki. The Ethics Committee of the Southern Medical University specifically confirmed that no informed consent was required because the data were to be analyzed anonymously.

### RNA isolation and quantitative real-time PCR

Total RNA was extracted with TRIzol Reagent (Invitrogen, Carlsbad, CA) according to the manufacturer’s instructions. cDNA was synthesized using the PrimeScript RT reagent kit (Promega, Madison, WI, USA). Quantitative real-time RT-PCR was carried out to detect the expression of MIER3 with the SYBR Premix EX Taq^TM^ (TaKaRa, Dalian, China) using an ABI 7500 Real-Time PCR system (Applied Biosystems, Foster City, USA). β-actin was used as an endogenous control. Fold changes were calculated through relative quantification (2^−△△Ct^). The primers for MIER3 were 5′-CTTTGGGTGGGACGGTAAATGCT-3′and 5′-CAGACGGTTGCTACACTGTT GGT-3′. The primers for β-actin were 5′-ACTCGTCATACTCCTGCT-3′ and 5′-GAAACTACCTTCAA CTCC-3′. To account for technical variability, the assay was performed in triplicate for each case.

### Immunohistochemistry and evaluation of MIER3 staining

MIER3 expression was examined using immunohistochemistry (IHC) in CRC and non-CRC paraffin-embedded sections. After the sections were deparaffined and rehydrated, they were submerged in EDTA antigenic retrieval buffer (pH = 8.2), microwaved, treated with 3% hydrogen peroxide in methanol to quench the endogenous peroxidase activity, incubated with 1% BSA to block the non-specific binding, incubated with antibodies against MIER3 (Abcam, Cambridge, UK) at a dilution of 1:150 overnight at 4 °C, and subsequently incubated for 30 minutes at 4 °C with HRP. Diaminobenzidine (DAB) was used for performing color reactions. For negative controls, the antibody was replaced by normal goat serum.

The immunohistochemically stained tissue sections were reviewed and scored separately by two pathologists blinded to the clinical parameters. Staining for MIER3 was assessed using a relatively simple, reproducible scoring method^16, 31^. The staining intensity was scored as 0 (negative), 1 (weak), 2 (medium), or 3 (strong). The extent of staining was scored as 0 (0%), 1 (1–25%), 2 (26–50%), 3 (51–75%) or 4 (76–100%), according to the percentage of positive staining areas in relation to the whole tumor area or the entire section for the normal sample. The sum of the intensity and extent scores was used as the final staining score (0–7) for MIER3. For statistical analysis, a final staining score of ≥3 was considered high, and scores of <3 were considered low for expression of MIER3.

### Western blot analysis and co-immunoprecipitation

Tissue and cells were lysed on ice in RIPA buffer with protease inhibitors. The protein concentration was measured using the Bradford protein assay (Bio-Rad Laboratories, Hercules, CA, USA). Equal amounts of protein were separated electrophoretically on 10% SDS/polyacrylamide gels and transferred onto a nitrocellulose membrane. After the membrane was blocked with 5% milk solution in tris-buffered saline with Tween (TBST) for 1 hour, it was probed using the following antibodies: anti-MIER3 rabbit polyclonal antibody (Abcam, Cambridge, Inc, ab127688 Recombinant fragment, corresponding to a region within amino acids 234–476 of human MIER3); anti-Sp1 (Abcam, Cambridge, Inc); anti-E-cadherin (Sigma-Aldrich); vimentin (Sigma-Aldrich); and ZO-1 (Abcam, Cambridge, Inc). Expression of these proteins was examined with horseradish peroxidase-conjugated anti-rabbit IgG (Cell Signaling Technology) and enhanced chemiluminescence (Amersham Biosciences Europe, Freiberg, Germany). An anti-*GAPDH* (Sigma-Aldrich) mouse polyclonal antibody was used to confirm equal loading of proteins.

For co-immunoprecipitation, cell lysates were prepared as described above from the CRC cell line SW620. The cell lysates were precleared by incubation with preblocked Protein A Sepharose beads (Zymed, San Francisco, CA, USA). Then, individual antibodies (MIER3, 1:500; Sp1, 1:1000; normal rabbit IgG) were added and incubated overnight at 4 °C. Complexes were then harvested with protein A Sepharose (GE Healthcare, Piscataway, NJ, USA) and briefly centrifuged. Bound proteins were separated with SDS/PAGE followed by visualization using western blotting.

### Vector construction, retroviral infection and transfection of colon cancer cells

The MIER3 construct was generated by subcloning PCR-amplified full-length human MIER3 cDNA into pEZ-Lv105. For deletion of MIER3, a preverified shRNA sequence (5′-GGACTATATGGATCGTTTA-3′) was cloned into a GV115 lentiviral vector to generate GV115-MIER3-shRNA. A scrambled shRNA oligo (5′-TTCTCCGAACGTGTCACGT-3′), which did not match any known human gene, was used as a control. The sequence of Sp1 was synthesized and subcloned into a pcDNA3.1 (Invitrogen, Shanghai, China) vector. Ectopic expression of Sp1 was achieved by transfection with pcDNA3.1-Sp1, and empty pcDNA3.1 vector was used as a control. Pre-verified and selected human small interfering RNAs (siRNAs) targeting Sp1 (5’-GCCAAUAGCUACUCAACUA-3’) and a human scrambled siRNA sequence possessing limited homology with human genes (used as a negative control) were synthesized. The expression level of Sp1 was detected by western blot analysis. Retroviral production, infection and transfection of colon cancer cells were performed as previously described^14^. Stable cell lines expressing MIER3 or shMIER3 were selected for 10 days with 0.5 mg/mL puromycin.

### Cell proliferation assay

Cell counting kit-8 (CCK-8, Dojindo, Rockville, USA) was used to evaluate the rate of cell proliferation according to the manufacturer’s instructions. The cells were seeded in 96-well plates at a density of 2 × 10^3^ per well and cultured 5 days. CCK-8 solution was added to each well. After the cells were incubated for 1 hour at 37 °C, the absorbance of each well was recorded at 450 nm and read on a microplate reader Victor (Enspire 2300 Multilabel Reader, PerkinElmer, Singapore).

### Colony formation assay

The cells were plated in 6-well plates (200/well) and maintained in RPMI 1640 containing 10% FBS for 2 weeks. The cells were washed twice with PBS, fixed with methanol and stained with 0.5% crystal violet. The number of colonies whose diameter was greater than 150 μm was counted under a microscope.

### Cell migration assay

Wound healing assays were performed to measure cell motility by measuring the movement of cells into a scraped, acellular area using a 200 μL pipette tip, and the wound closure was observed after 0 and 48 hours. Photographs were taken to assess the level of migration in each group of transfected cells. The migration was quantified by counting the total number of cells that migrated toward the original wound field.

### Cell invasion assay

Matrigel-coated chambers (BD Biosciences, San José, CA, USA) containing 8 µm pores were used for invasion assays. Cells were seeded into the top chambers (coated in Matrigel) with serum-free medium (2 × 10^5^ cells). The bottom chamber of the Transwell was prepared with culture media containing 10% FBS as a chemo-attractant. After these chambers were incubated at 37 °C for 48 hours, non-invaded cells on the top of the Transwell were scraped off with a cotton swab. The successfully translocated cells were fixed with 10% formalin. Then, these cells were stained with 0.1% crystal violet for 30 minutes and counted under a light microscope.

### Animal experiments

Balb/C-nu/nu athymic nude mice (4–6 weeks old) were obtained from the Laboratory Animal Centre of Southern Medical University, which is certified by the Guangdong Provincial Bureau of Science. All mice were housed under specific pathogen-free conditions. Xenograft tumors were generated by the subcutaneous injection of 2 × 10^6^ suspended cells into the armpits of mice. The tumor size was measured using a digital caliper every four days. After monitoring for 28 days, mice were sacrificed by cervical dislocation, and tumors were dissected. Tumor volume was calculated as follows: volume = (D × d^2)^/2, where D refers to the longest diameter and d refers to the shortest diameter.

To evaluate *in vivo* metastasis, 2 × 10^6^ CRC cells or control cells were injected into the tail veins of nude mice. Animal health status was determined every three days. At the time of euthanasia, the lungs were removed by dissection away from adjacent organs and fixed with 10% neutral buffered formalin. Subsequently, the consecutive tissue sections were obtained and stained with hematoxylin-eosin to observe the metastatic nodules of the lungs under a microscope. All animal experiments were conducted in strict accordance with the principles and procedures approved by the Committee on the Ethics of Animal Experiments of Southern Medical University.

### Statistical analysis

All statistical analyses were performed using the SPSS 16.0 statistical software package. In at least three independent experiments, the data were presented in terms of the mean ± SD. The differences between variables were assessed using three statistical tests: theχ^2^ test, Fisher’s exact test, or one-way ANOVA. The survival curves were plotted using the Kaplan-Meier method and compared using the log-rank test. Multivariate survival analysis was performed on all parameters that were found to be significant in univariate analysis using the Cox regression model. A *p* value less than 0.05 was considered statistically significant.
